# Exploration of adverse events associated with risdiplam use: Retrospective cases from the US Food and Drug Administration Adverse Event Reporting System (FAERS) database

**DOI:** 10.1371/journal.pone.0298609

**Published:** 2024-03-01

**Authors:** Lurong Yu, Limei Liu

**Affiliations:** 1 College of Traditional Chinese Medicine of Chongqing Medical University, Chongqing, China; 2 Pharmacy Department of Chongqing YouYou BaoBei Women’s and Children’s Hospital, Chongqing, China; Iowa State University, UNITED STATES

## Abstract

Risdiplam is a new drug for treating spinal muscular atrophy (SMA). However, pharmacovigilance analyses are necessary to objectively evaluate its safety—a crucial step in preventing severe adverse events (AEs). Accordingly, the primary objective of the current study was to examine the AEs associated with risdiplam use based on real-world data obtained from the US Food and Drug Administration Adverse Event Reporting System (FAERS) database. More specifically, we examined incidents reported between the third quarter of 2020 and the second quarter of 2023. The imbalance of risdiplam-related AEs was evaluated by computing the reporting odds ratio. A total of 5,406,334 reports were thoroughly reviewed. By removing duplicate reports, we identified 1588 reports in which risdiplam was the main suspected drug whose use was accompanied by 3470 associated AEs. Among the included AEs, 703 were categorized as serious and 885 as non-serious. Risdiplam use induced AEs across 18 organ systems, resulting in 130 positive signals. Notably, we detected new AE signals, including cardiac arrest, nephrolithiasis, tachycardia, loss of libido, and elevated hepatic enzyme activities; however, no ophthalmologic toxicity was reported. Although these new adverse reaction signals associated with risdiplam have been defined, long-term clinical studies are needed to confirm these findings. Nevertheless, our findings provide a valuable reference for improving the clinical management of SMA.

## Introduction

Spinal muscular atrophy (SMA) is a genetic autosomal recessive disease, which means that it requires two copies of the mutated gene to be inherited. It is estimated to occur in approximately one of every 6,000–10,000 live births [1]. The mutated survival motor neuron 1 (*SMN1*) gene, encoding SMN, is the primary factor responsible for SMA development [2]. The disease is distinguished by the gradual weakening and immobility of muscles near the centre of the body and is categorized into types 1, 2, 3, and 4 [3], which align with the appearance of symptoms in the first 6 months, 7–18 months, > 18 months, and 20–30 years, respectively [4]. Infants with type 1 SMA are typically unable to sit independently [5] and, if left untreated, rarely survive beyond their second year of life [5]. Although individuals with type 2 SMA may be able to sit or stand with assistance, they lack the ability to walk independently. Type 3 SMA is characterized by symptoms that develop after 18 months [5], and type 4 represents a milder form that occurs in adults and does not significantly impact life expectancy [6]. Meanwhile, by modifying the splicing of survival motor neuron 2 (SMN2) pre-mRNA, risdiplam upregulates SMN protein levels [4]. Hence, this medication elicits clinical improvements in motor function among individuals with SMA, including children, teenagers, and adults [7]. Furthermore, it has been approved for SMA treatment in various countries [6]. Particularly, on 7 August 2020, the United States Food and Drug Administration (FDA) approved risdiplam as a treatment option for patients with SMA who are two months or older.

During the clinical phase III trials of risdiplam, the most frequently reported adverse events (AEs) were fever, diarrhoea, skin rash, ulcers in the mouth and oral area, urinary tract infection, and joint pain [8]. According to Masson et al. [9], upper respiratory tract infection was the most frequently reported AE, affecting 22 infants (54%). However, pneumonia was the most common serious AE, occurring in 16 infants (39%), followed by respiratory distress, affecting 3 infants (7%). Clinical trials have provided information regarding the long-term effectiveness and safety of risdiplam. However, the relatively small sample size has resulted in most AEs being associated with various organs; additionally, there is a lack of extensive data on the effectiveness and safety of risdiplam.

The openly accessible US Food and Drug Administration Adverse Event Report System (FAERS) database provides information about a wide range of AEs associated with drugs that are reported by drug manufacturers, patients, and healthcare professionals [10]. Indeed, its global reach has resulted in collection of information from reports from the United States as well as various other countries [10]. In this study, a comprehensive risk profiling of risdiplam was conducted using information from the FAERS database to provide a valuable reference for improving the clinical management of SMA.

## Materials and methods

### Data source

The data used in this study were obtained from the publicly available FAERS database. The FAERS data files contained seven datasets—patient demographics and administration (DEMO), drug details (DRUG), records of AEs (REAC), patient outcomes (OUTC), sources of reports (RPSR), start and end dates of therapy for the reported drugs (THER), and indications for drug usage (INDI) [11]. To conduct this study, we extracted all data in the ASCII format, encompassing the timeframe from Q3 of 2020 to Q2 of 2023. Subsequently, we imported the data into the SAS 9.4 software for cleaning and analysis. This retrospective study analysed anonymized publicly available human data. Accordingly, the requirement for patient consent was waived, and the study was deemed exempt from ethical review by the Ethics Committee of Chongqing Youyoubaobei Women and Children’s Hospital on 13 July 2023.

### Data processing

Following the FDA-recommended duplicate reporting method, the DEMO files were processed by selecting and sorting them first using CASEID, then using FDA_DT, and finally using PRIMARYID. To ensure correctness, the most recent FDA_DT (date the FDA picked up the case) was selected when it matched the CASEID (FAERS case identification number) [11]. In the case when CASEID and FDA_DT were identical, the report with the higher PRIMARYID (a unique identifier for FAERS reports) was chosen [11]. Notably, the AEs in the FAERS are labelled using the preferred terms (PTs) from the International Dictionary of Medical Terms (MedDRA). MedDRA undergoes yearly updates in March and September and includes adjustments to the PTs and the system organ classification (SOC). Accordingly, in the present study, the latest MedDRA version (MedDRA, version 26.0) was used to correct the PTs and obtain the latest SOC and PT information from the REAC files.

### Targeted drug screening

In the FAERS database, the DRUGNAME field represents the name of the drug, while the PROD_AI field represents the product composition. The PROD_AI field was added to the FAERS database in the third quarter of 2014. Regarding drugs approved for marketing after this period, the target drug can be screened based on PROD_AI. Conversely, for drugs approved before the third quarter of 2014, screening is based on DRUGNAME and PROD_AI, with reports limited to "primary suspicion (PS).” Risdiplam was approved by the FDA on 7 August 2020, making it eligible for screening via the PROD_AI field, which is indexed with “Risdiplam” as the keyword.

### Statistical analysis

Analysing disproportionality involves the utilization of statistical methods to compare the reporting rates of the study drug with those of all drugs combined in the spontaneous reporting database [12]. Following the principles of non-proportional analysis, the reporting odds ratio (ROR) was utilized to assess the relationships between the drugs and selected AEs [11]. A sign of imbalance was identified when the minimum value of the 95% confidence interval (CI) for ROR was > 1 and it was supported by at least three pieces of evidence [10, 13, 14]. The calculations were derived from the 2×2 contingency table (Table 1).

**Table 1:** The 2 × 2 contingency table.

Drug category	Target adverse event	Non-target adverse event
Risdiplam	a	b
Non-risdiplam	c	d


$$ROR=(a/b)/(c/d),\;95\%\;CI=e^{ln(ROR)\pm 1.96(1/a+1/b+1/c+1/d)ˆ0.5}$$ (1)

Descriptive statistics were executed to summarize the demographic traits of the patients and clinical features retrieved from the FAERS database. A p-value < 0.05 was used to establish statistical significance, with a 95% CI applied.

## Results

### General characteristics

**Fig 1:**
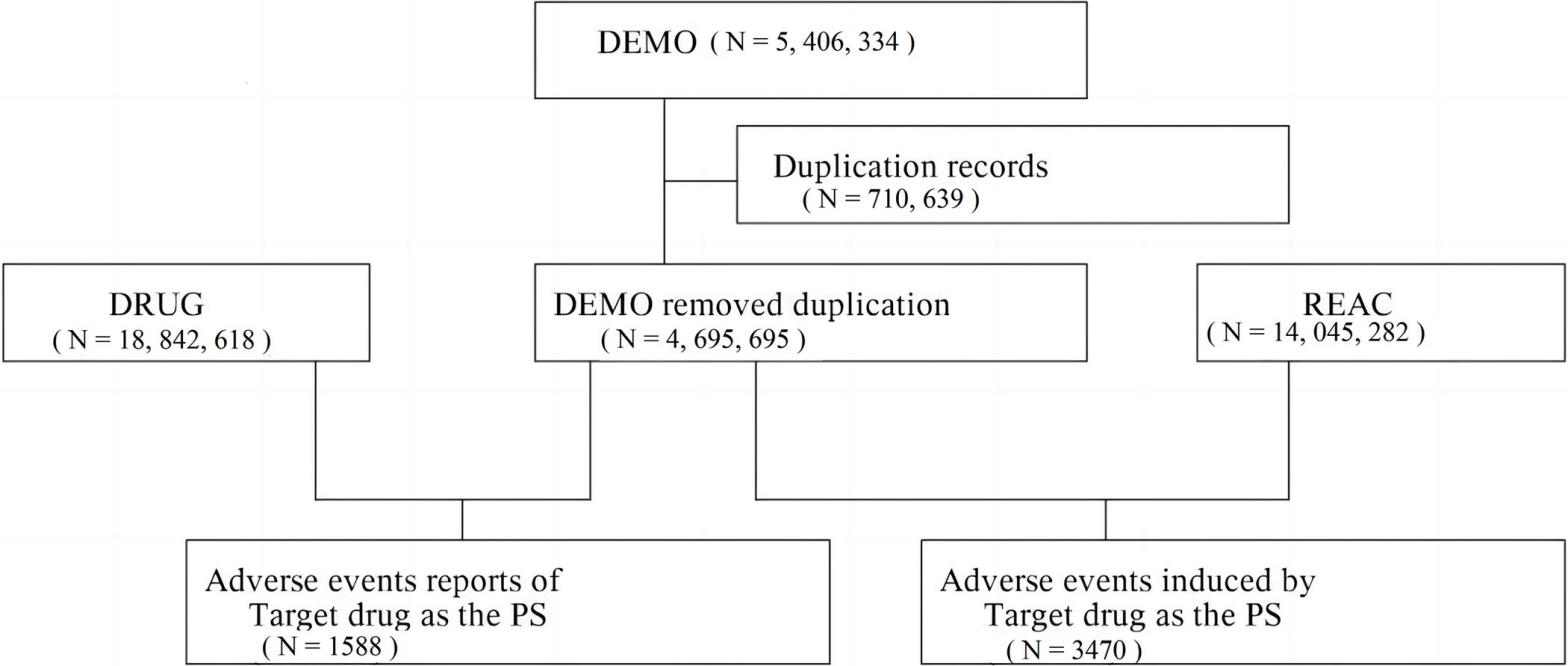
Flowchart depicting the selection of the study population. DEMO, patient demographics; DRUG, administration drug details; REAC, records of adverse events.

During the study period, the FAERS database reviewed 5,406,334 reports. After excluding duplicates, we identified 1588 reports linked to risdiplam as the primarily suspected drug, along with 3470 related AEs. Fig 1 demonstrates the steps involved in reviewing the reports. Risdiplam-related reports are listed in Table 2, providing details about the clinical features. Among the included reports, 703 cases were categorized as serious and 885 as non-serious. Females accounted for 47.54% of the cases. In addition, most reported cases with known ages involved individuals < 45 years of age. The USA reported the highest number of AEs, at 75.94%, followed by the UK (4.09%), the Netherlands (2.58%), India (1.95%), and Germany (1.83%). The annual number of reports showed an increasing trend from 2020 to 2023.

**Table 2:** Clinical characteristics of the included cases treated with risdiplam and the associated adverse events.

Characteristic	Counts (%)
Number of events	1588
Sex	
Female	755 (47.54)
Male	552 (34.76)
Unknown	281 (17.70)
Age (years)	
< 18	355 (22.36)
≥ 18 and < 45	288 (18.14)
≥ 45 and < 65	96 (6.05)
≥ 65	22 (1.38)
Missing	827 (52.08)
Mean (SD)	23.16 (18.59)
Median (Q1, Q3)	19.00 (7.00, 36.00)
Reporting year	
2020	88 (5.54)
2021	517 (32.56)
2022	540 (34.01)
2023 (two quarters)	443 (27.90)
Type of reporter	
Consumer	1006 (63.35)
Physician	401 (25.25)
Pharmacist	180 (11.34)
Missing	1 (0.06)
Reporting country (Top 5)	
United States of America	1206 (75.94)
United Kingdom	65 (4.09)
Netherlands	41 (2.58)
India	31 (1.95)
Germany	29 (1.83)
Types of report	
Non-serious	885 (55.73)
Serious	703 (44.27)
Outcome	
Life-threatening	29 (1.83)
Hospitalization–Initial or Prolonged	304 (19.14)
Disability	40 (2.52)
Death	127 (8.00)
Congenital Anomaly	3 (0.19)
Required Intervention to Prevent Permanent Impairment/Damage	0 (0.00)
Other serious and important medical events	309 (19.46)
Time to event onset (days)	
0–30 d	156 (9.82)
31–60 d	40 (2.52)
61–90 d	23 (1.45)
91–120 d	20 (1.26)
121–150 d	14 (0.88)
151–180 d	13 (0.82)
181–360 d	42 (2.64)
360 d <	93 (5.86)
Unknown N (Missing) Mean (SD) Median (Q1, Q3)	1187 (74.75) 401 (1187) 238.25 (707.13) 64.00 (10.00, 333.00)

### Time to event onset

**Fig 2:**
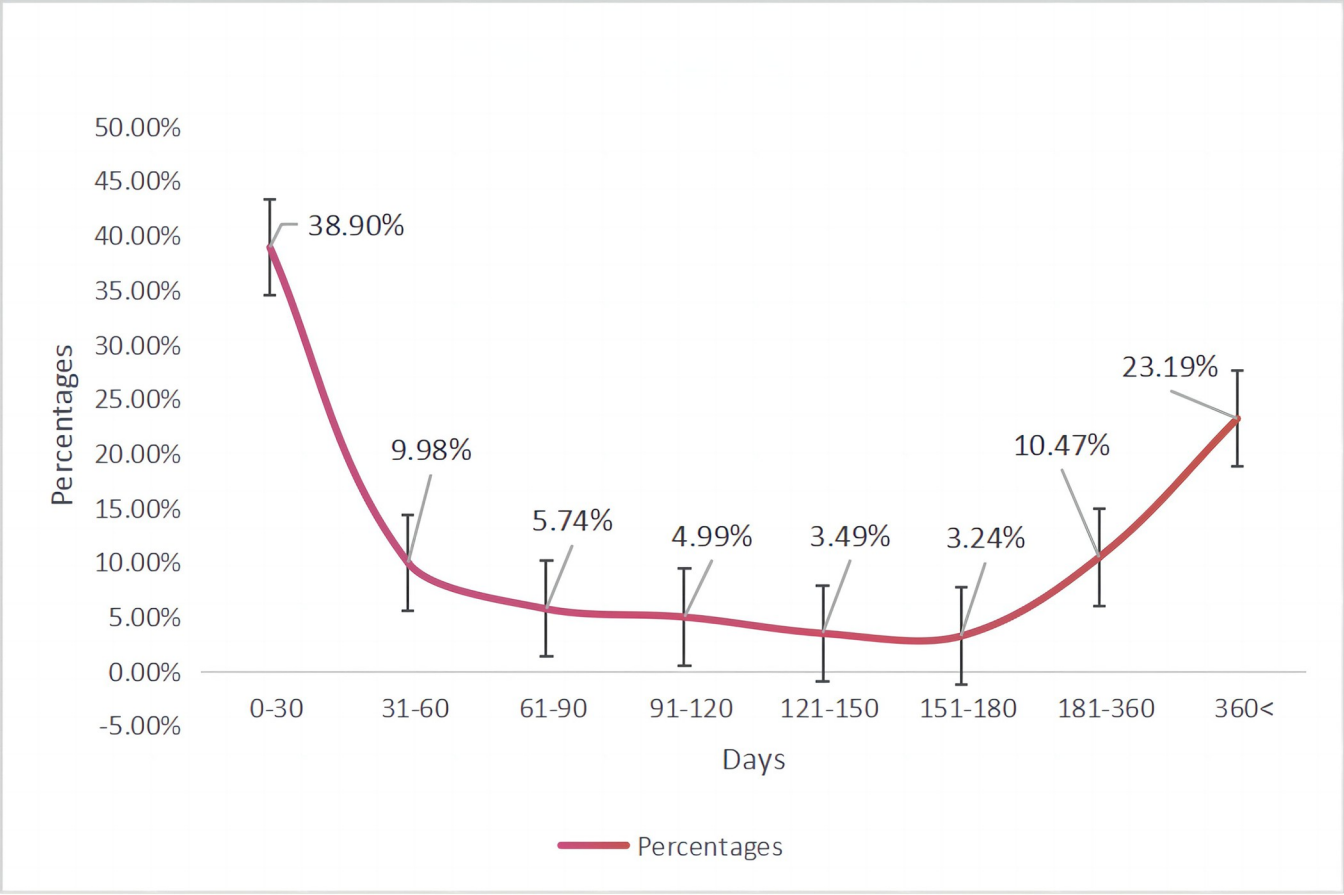
Time to onset of risdiplam-related AEs. P (%) = (n/401) × 100%; n is the frequency of each time period.

Information regarding for the reports that included AE onset (*n* = 401) is provided in Table 2. The median duration from the beginning of the events was 64 days (range: 10–333 days). The time when the AEs began is illustrated in Fig 2. Most AEs occurred within the first month (38.9%), followed by those occurring after one year of risdiplam treatment (23.19%).

### Signal detection

**Fig 3:**
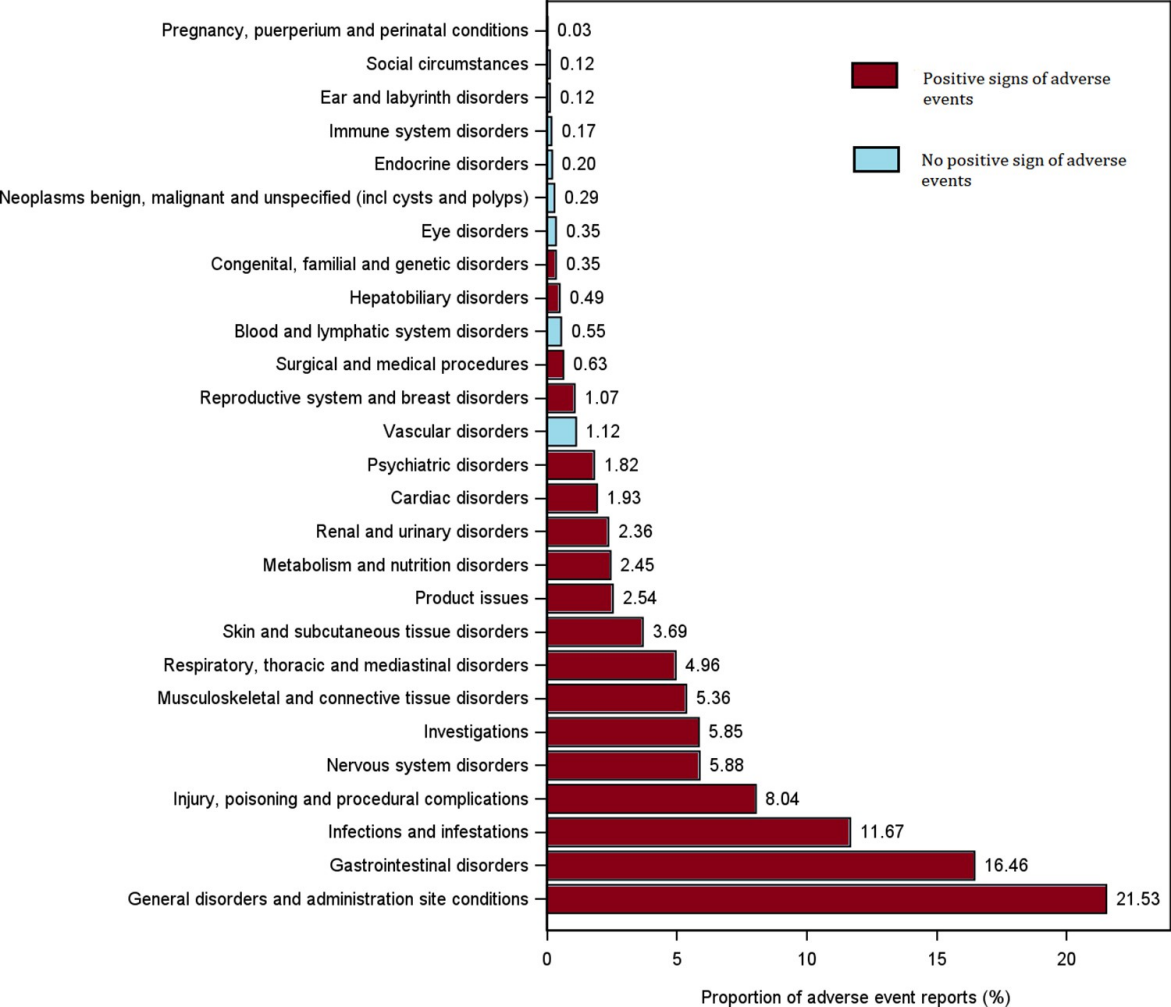
Proportion of adverse events under system organ classification (SOC).

Fig 3 presents the proportion of AEs categorized under the SOC. AEs were most commonly observed in the general disorders and administration site conditions category (21.53%), followed by those in gastrointestinal disorders (16.46%), infections and infestations (11.67%), injury, poisoning, and procedural complications (8.04%), and nervous system disorders (5.88%). Table 3 shows the distribution of positive signals under SOC and the number of AE reports by sex, age group, and reporting country. We found that risdiplam induced AEs across 18 organ systems, resulting in the detection of 130 positive signals. Across sex and age groups, general disorders and administration site conditions, gastrointestinal disorders, and infections and infestations were among the most prevalent conditions. However, notable variations were detected among countries. Specifically, in the Netherlands, the leading three were investigations, infections and infestations, and metabolism and nutrition disorders.

**Table 3:** Distribution of reported adverse events by sex, age, and reporting country under the classification of systemic diseases.

System organ classification (SOC)	Number of signals	Counts (N)	Sex (Count, n)		Age (Count, n)				Reporting country (Count,)				
			Female	Male	< 18	≥ 18, < 45	≥ 45, < 65	≥ 65	USA	UK	Netherlands	India	Germany
General disorders and administration site conditions	11	747	346	239	135	139	37	9	613	26	1	15	13
Gastrointestinal disorders	21	571	281	196	134	104	39	6	457	20	1	0	10
Infections and infestations	21	405	214	152	170	88	27	9	224	24	14	12	13
Injury, poisoning and procedural complications	12	279	141	86	49	58	18	3	250	8	1	0	6
Nervous system disorders	8	204	101	75	69	38	13	2	145	8	4	0	7
Investigations	9	203	108	79	54	58	20	1	129	12	15	3	11
Musculoskeletal and connective tissue disorders	6	186	86	69	22	47	14	1	158	8	1	1	4
Respiratory, thoracic and mediastinal disorders	10	172	88	68	78	28	9	1	82	8	3	7	7
Skin and subcutaneous tissue disorders	4	128	67	41	46	14	13	0	90	6	0	1	3
Product issues	7	88	45	27	13	24	12	1	81	1	1	0	0
Metabolism and nutrition disorders	4	85	44	38	20	31	9	3	46	4	14	0	2
Renal and urinary disorders	7	82	48	26	23	20	10	1	41	3	8	1	4
Cardiac disorders	3	67	37	26	26	16	3	5	28	5	2	2	4
Psychiatric disorders	1	63	30	21	13	16	2	1	57	2	0	1	0
Vascular disorders	0	39	16	17	10	7	6	1	25	3	3	0	0
Reproductive system and breast disorders	2	37	26	6	3	18	3	0	19	2	0	0	4
Surgical and medical procedures	2	22	8	14	6	2	0	0	6	1	0	0	3
Blood and lymphatic system disorders	0	19	12	5	6	4	6	0	4	4	3	2	1
Hepatobiliary disorders	1	17	11	5	6	3	3	0	5	4	0	1	2
Congenital, familial, and genetic disorders	1	12	2	4	3	2	0	0	10	0	1	0	0
Eye disorders	0	12	5	6	6	4	0	0	6	1	0	0	1
Neoplasms benign, malignant and unspecified (incl. cysts and polyps)	0	10	6	3	1	1	2	0	2	3	0	0	0
Endocrine disorders	0	7	3	3	3	0	1	0	4	0	0	0	0
Immune system disorders	0	6	2	2	1	2	0	0	5	0	1	0	0
Ear and labyrinth disorders	0	4	2	2	1	3	0	0	1	1	0	0	0
Social circumstances	0	4	2	2	0	2	0	0	3		0	0	0
Pregnancy, puerperium and perinatal conditions	0	1	1	0	0	0	0	0	1		0	0	0
	130	3470											

Note: SOC, system organ class; USA, United States of America; UK, United Kingdom

Table 4 lists the top five AEs regarding signal strength at the PT level by sex, age group, and reporting country that produced a positive signal. In the sex subgroup, product container issues was mentioned by males and female. In addition, dermatitis diaper, maternal exposure timing unspecified, loss of libido, and bladder pain were reported in the female group, while tracheostomy, mechanical ventilation, respiratory tract infection viral, and motor dysfunction were reported in the male group. In the age subgroups, product container issues were reported in all groups < 65 years of age. Meanwhile, kidney stones were reported in two groups—age < 18 years and age ≥ 45 years but less than 65 years. Among the different age groups, the greatest AE signal intensities were assigned to bronchial obstruction (age < 18), product container issues (age ≥ 18 years but less than 65 years), and diarrhoea (age ≥ 65 years).

**Table 4:** Subgroup analysis of the top five signal intensities producing positive signals for adverse events at the PT level according to sex, age, and reporting countries.

Category	PT	n	ROR	95% CI Lower	95% CI Upper
Sex					
Female	Dermatitis diaper	4	248.79	90.73	682.19
	Maternal exposure timing unspecified	9	75.71	39.11	146.59
	Product container issue	14	71.95	42.34	122.27
	Loss of libido	3	34.44	11.05	107.37
	Bladder pain	3	33.21	10.66	103.53
Male	Tracheostomy	4	143.08	52.68	388.57
	Mechanical ventilation	5	133.34	54.60	325.65
	Respiratory tract infection viral	4	108.78	40.22	294.19
	Product container issue	6	65.37	29.12	146.75
	Motor dysfunction	6	31.85	14.24	71.27
Age (years)					
< 18	Bronchial obstruction	3	74.13	22.07	248.97
	Product container issue	6	70.99	30.18	167.02
	Dermatitis diaper	4	47.22	16.93	131.71
	Asphyxia	4	31.48	11.45	86.55
	Nephrolithiasis	6	23.31	10.26	52.95
≥18 and < 45	Product container issue	7	257.97	118.32	562.42
	Product packaging quantity issue	4	80.07	29.53	217.09
	Exposure via skin contact	3	39.58	12.63	124.05
	Diverticulitis	4	38.02	14.13	102.28
	Product leakage	3	30.56	9.77	95.60
≥ 45 and < 65	Product container issue	7	257.97	118.32	562.42
	Exposure via skin contact	4	80.07	29.53	217.09
	Nephrolithiasis	3	39.58	12.63	124.05
	Hypokalaemia	4	38.02	14.13	102.28
	Muscular weakness	3	30.56	9.77	95.60
≥ 65	Diarrhoea	3	5.96	1.83	19.34
Country					
USA	Dermatitis diaper	5	160.88	65.51	395.09
	Maternal exposure timing unspecified	9	96.06	49.46	186.58
	Product container issue	26	61.45	41.61	90.75
	Respiratory tract infection viral	4	55.68	20.71	149.69
	Intercepted medication error	8	50.27	24.98	101.17
UK	Spinal disorder	5	417.58	161.64	1078.79
	Lower respiratory tract infection	10	12.06	6.35	22.93
	Illness	4	9.30	3.44	25.14
	Urinary tract infection	3	6.18	1.97	19.41
	Death	6	2.54	1.12	5.74
Netherlands	Hypokalaemia	14	107.92	59.48	195.78
	Electrocardiogram QT prolonged	5	52.71	20.98	132.44
	Pneumonia	3	8.70	2.73	27.71
India	Pneumonia	6	33.13	13.94	78.70
	Death	13	4.96	2.61	9.43
Germany	Drug ineffective	3	4.27	1.35	13.50

Note: Age ≥ 65 years and adverse events reported in the Netherlands, India, and Germany yielded fewer than five positive signals, summarized according to the actual situation.

Reporting odds ratio (ROR), confidence interval (CI), preferred term (PT)

Table 5 presents the positive signal-producing AEs at the PT level, categorized by sex, age group, and reporting country, with the top five reported events showcased. Diarrhoea was reported in the different sex and age groups. In the sex subgroups, the most frequent reports were of diarrhoea in males and asthenia in females. In the different age groups, pneumonia (age < 18 years), Asthenia (age ≥ 18 years but less than 45 years), and diarrhoea (age ≥ 45 years) were the most frequently reported signal-producing AEs. In the country groups, diarrhoea (USA), lower respiratory tract infection (UK), hypokalaemia (Netherlands), death (India), and drug ineffectiveness (Germany) were the most commonly reported conditions.

**Table 5:** Five most frequent adverse events that generated positive signals at the PT level based on subgroup analyses according to sex, age, and reporting country.

Sex (Count, n)				Age (years; count, n)							
Preferred terms (PT)	Male	PT	Female	PT	< 18	PT	≥ 18 and < 45	PT	≥ 45 and < 65	PT	≥ 65
Diarrhoea	58	Asthenia	59	Pneumonia	45	Asthenia	26	Diarrhoea	10	Diarrhoea	3
Pneumonia	30	Diarrhoea	47	Diarrhoea	23	Fatigue	25	COVID-19	7		
Asthenia	30	Fatigue	45	Death	23	Diarrhoea	19	Muscular weakness	6		
Fatigue	27	Pneumonia	35	Pyrexia	23	COVID-19	17	Constipation	4		
Pyrexia	25	Muscular weakness	25	Vomiting	21	Muscular weakness	14	Product container issue	4		
**Reporting country (Count,n)**											
PT	USA	PT		UK	PT		Netherlands	PT	India	PT	Germany
Diarrhoea	118	Lower respiratory tract infection		10	Hypokalaemia		14	Death	13	Drug ineffective	3
Asthenia	104	Death		6	Electrocardiogram QT prolonged		5	Pneumonia	6		
Fatigue	85	Spinal disorder		5	Pneumonia		3				
Muscular weakness	51	Illness		4							
Pneumonia	39	Urinary tract infection		3							

Note: Age ≥ 65 years and adverse events reported in the Netherlands, India, and Germany yielded fewer than five positive signals, summarized according to the actual situation.

Table 6 presents the newly identified AEs that were reported at the PT level with a minimum occurrence of ten and a detected positive signal. The three most frequently reported events in order were asthenia, fatigue, and death. Furthermore, cardiac disorders were associated with three different AEs: increased heart rate, tachycardia, and cardiac arrest. Similarly, two AEs were associated with renal and urinary disorders: renal and urinary renal and urinary disorders.

**Table 6:** Positive signals generated by newly reported adverse events reported at least ten times.

PT	N	ROR	95% CI Lower	95% CI Upper	PT	N	ROR	95% CI Lower	95% CI Upper
Asthenia	113	6.24	5.17	7.52	Paraesthesia	19	2.46	1.56	3.85
Fatigue	92	2.08	1.69	2.56	Respiratory disorder	19	12.82	8.16	20.14
Death	69	1.41	1.11	1.79	Respiratory failure	18	5.62	3.53	8.93
Muscular weakness	54	10.82	8.27	14.16	Tachycardia	17	3.91	2.43	6.30
COVID-19	46	1.54	1.15	2.06	Abdominal distension	17	3.45	2.14	5.55
Abdominal pain upper	35	3.43	2.46	4.79	Gastric disorder	16	7.48	4.57	12.22
Product storage error	35	4.98	3.57	6.94	Hypokalaemia	15	6.63	3.99	11.02
Gastrointestinal disorder	34	7.40	5.27	10.37	Myalgia	15	2.06	1.24	3.43
Abdominal discomfort	31	3.09	2.17	4.40	Accidental exposure to product	14	2.08	1.23	3.52
Weight decreased	30	1.90	1.33	2.73	Cardiac arrest	13	3.97	2.30	6.85
Product container issue	26	68.16	46.20	100.58	Flatulence	12	4.50	2.55	7.93
Heart rate increased	25	4.81	3.25	7.13	Product quality issue	12	3.45	1.96	6.08
Nephrolithiasis	23	8.60	5.71	12.97	Respiratory tract infection	11	8.59	4.75	15.53
Ill-defined disorder	22	7.28	4.78	11.07	Hepatic enzyme increased	11	2.84	1.57	5.14
Influenza	21	4.06	2.64	6.23	Photosensitivity reaction	11	13.44	7.43	24.32
Dysphagia	20	4.78	3.08	7.43	Viral infection	10	6.82	3.67	12.70
Abdominal pain	20	1.74	1.12	2.69	Respiratory syncytial virus infection	10	19.13	10.27	35.64
Sepsis	20	3.70	2.38	5.74	Muscle twitching	10	10.17	5.46	18.93
Weight increased	19	1.60	1.02	2.51	Urinary retention	10	6.34	3.41	11.80

Note: New adverse events = adverse events outside the labelling of risdiplam (https://www.drugfuture.com/fda/drugview/213535) and [1, 8, 9].

Reporting odds ratio (ROR), confidence interval (CI); preferred terms (PT)

## Discussion

Maintaining a constant awareness of the potential signs of harmful drug reactions (ADRs) and consistently reporting suspected drug-related reactions after drug approval are crucial for evaluating the safety of a medication and achieving an equilibrium between its benefits and risks when making clinical decisions [15]. Herein, to prevent significant drug safety issues, risdiplam—recently approved for treating SMA—was subjected to pharmacovigilance analyses to objectively evaluate its safety. We identified 130 positive AE signals related to risdiplam, involving several systems, such as general disorders and administration site conditions, gastrointestinal disorders, infections and infestations, and nervous system disorders. The AEs associated with risdiplam that were observed in previous clinical studies [1, 8, 9] are consistent with our findings (Tables 4 and 5) and include diarrhoea, pyrexia, vomiting, lower respiratory tract infection, urinary tract infection, and constipation. The top five most frequently reported AEs that generated positive signals varied by sex, age group, and reporting country. Diarrhoea and pneumonia were the most frequently reported AEs according to sex, age group, and reporting country.

Non-clinical studies have shown that risdiplam, which modifies the splicing of SMN2 mRNA, also affects other mRNA splice targets such as Forkhead Box M1 (FOXM1) and MAP kinase-activating death domain protein (MADD) [16–18]. Our study revealed that females reported decreased libido, generating the fourth strongest signal, which was not reported by males. However, we did not observe other AEs that generated a favourable indication and were of significance to reproduction. In a previous study, seminiferous tubule degeneration was noted in rats, with a 50% reversal rate of germ-cell degeneration in the testes observed in rats exposed to risdiplam [16]. Accordingly, it is expected that any effects on the male reproductive system caused by these SMN2 mRNA-splicing modifiers would be reversible in humans [16].

Furthermore, we identified certain novel negative occurrences that resulted in positive signals, including cardiac arrest, tachycardia, urinary retention, flatulence, photosensitivity reaction, and elevated hepatic enzyme activities. Although the precise underlying mechanisms remain unclear, this discovery highlights the need for clinicians to not exclude risdiplam when considering the cause of these AEs.

Risdiplam can induce off-target effects when splicing or replacing the defective *SMN 1* gene [19]. These off-target effects are believed to be responsible for AEs [19] and may involve misidentification and non-specific recognition. While risdiplam primarily impacts *SMN2* exon 7 to treat SMA, it can also affect *MBNL1* (muscleblind-like 1) exon 5, *DST* (i.e., BPAG1, bullous pemphigoid antigen 1), *TEAD1* (transcriptional enhancer activator domain 1), and *THOC5* (THO complex 5). Hence, the off-target effects of risdiplam can lead to recognition errors, as these impacted exons have structures similar to those of their intended targets. For example, *MBNL1* exon 5 has a G residue in its last position, as do *DST*, *TEAD1*, and *THOC5* [20]. MBNL1 plays a crucial role in controlling RNA metabolism; *MBNL1* exon 5 encodes a signal that determines the location of MBNL1 in the cytoplasm and is necessary for general gene expression regulation [21]. Meanwhile, abnormal transcripts that contain repetitive sequences and trap MBNL1 in the cell nucleus have been linked to ankylosing muscular dystrophy [22]. *DST*, *TEAD1*, and *THOC5* encode proteins involved in microtubule organization, transcription, and RNA metabolism, respectively. Accordingly, disruption of their expression or function is associated with neurological disorders [23]. Additionally, we observed negative effects within specific organs, including muscle weakness, myalgia, muscle twitching, and paraesthesia. Therefore, clinicians must consider these potential AEs when considering the use of risdiplam. In addition to its non-target effects on the disease targets *FOXM1* and *MADD* [24], risperdal also targets the 5′-splice site and GA-rich sequence, located 24 nucleotides upstream, for SMA treatment [25]. *FOXM1* encodes a crucial regulator of the cell cycle. That is, FOXM1 is necessary for cell division and is predominantly located within rapidly dividing cells, such as those in the gastrointestinal tract, male germ cells, skin, and haematopoietic progenitors in the bone marrow. Additionally, MADD is associated with apoptosis. These observations indicate that risperdal, a non-specific drug, targets FOXM1 and MADD, thereby disrupting the cell cycle, inducing micronucleus formation, and initiating apoptosis [24].

Witte et al. [26] showed that drugs modifying *SMN2* splicing produce off-target effects, including thrombotic microangiopathy, which can cause albuminuria and haematuria. Moreover, Qi et al. [27] noted a potential correlation between FOXMl targets and albuminuria, which is consistent with the urinary conditions detected in the current study, such as urinary retention, Nephrolithiasis. It is, thus, necessary to closely monitor urinary system functioning in patients with renal conditions prescribed risdiplam and consider potential dosage adjustment. Moreover, additional analysis regarding the safety and efficacy of risdiplam use for patients with renal impairment is warranted.

Wu et al. [28] proposed a potential association between *MADD* and increased vulnerability to diastolic heart failure. They argue that *MADD* is crucial in balancing the effects of TNF-⍺ [29]. The immediate effect on action potential duration, peak Ca^2+^ transient amplitude, and the rate of Ca^2+^ decline is detectable with low levels of TNF-⍺, varying between 200 and 500 U/mL [28]. In the present study, we identified safety signals related to tachycardia, cardiac arrest, and prolonged QT. These findings indicate that it is essential to monitor cardiac function during clinical use and consider potential dose adjustment. Although long-term, nonclinical safety studies in monkeys revealed off-target retinal effects [30], Robert et al. [31] verified that risdiplam does not harm the eyes of children or adults. However, notably, the label for risdiplam mentions the presence of retinal toxicity observed in animal studies, the specific mechanisms for which remain unclear.

Furthermore, we found that the signal intensity and frequency of AEs related to product leakage, product container issues, and product container seal problems were relatively high. This may be due to negligence in the usage of risdiplam and management of its dosage. This highlights the need for improved education and standard management of risdiplam use to prevent AEs resulting from product misuse. In addition, the patients reporting these AEs primarily included children under 18 years of age, the main incidence group being infants within 18 months of birth. Accordingly, healthcare professionals must closely monitor children prescribed this drug for potentially severe AEs.

## Conclusion

Through a comprehensive analysis of reports from the FAERS database, we demonstrated the potential risk signal and timing of AEs associated with risdiplam use. Major AEs, such as tachycardia, cardiac arrest, and nephrolithiasis, may occur; however, no cases of ophthalmologic toxicity were reported. Hence, clinicians must remain cautious regarding the potential occurrence of serious AEs associated with risdiplam, such as cardiac arrest, loss of libido, and elevated hepatic enzyme activities. Moreover, patients must be properly educated about potential AEs, and their physical well-being must be monitored to optimize their quality of life. These steps contribute to effective disease management. However, long-term clinical studies are needed to confirm our findings concerning the AEs associated with risdiplam use.
